# Cross sectional study of serum selenium concentration and esophageal squamous dysplasia in western Kenya

**DOI:** 10.1186/s12885-017-3837-9

**Published:** 2017-12-08

**Authors:** Natalie R. Pritchett, Stephen L. Burgert, Gwen A. Murphy, John D. Brockman, Russell E. White, Justus Lando, Robert Chepkwony, Mark D. Topazian, Christian C. Abnet, Sanford M. Dawsey, Michael M. Mwachiro

**Affiliations:** 1 National Cancer Institute, Bethesda, MD USA; 2 Tenwek Hospital, Bomet, Kenya; 3 University of Missouri Research Reactor Center, Columbia, MO USA; 4 Mayo Clinic, Rochester, Minnesota USA

**Keywords:** Esophageal, Squamous cell carcinoma, Squamous dysplasia, Selenium, Kenya

## Abstract

**Background:**

Low serum selenium status has been associated with increased risk of esophageal squamous cell carcinoma (ESCC). East Africa is a region of high ESCC incidence and is known to have low soil selenium levels, but this association has not previously been evaluated. In this study we assessed the association of serum selenium concentration and the prevalence of esophageal squamous dysplasia (ESD), the precursor lesion of ESCC, in a cross-sectional study of subjects from Bomet, Kenya.

**Methods:**

294 asymptomatic adult residents of Bomet, Kenya completed questionnaires and underwent endoscopy with Lugol’s iodine staining and biopsy for detection of ESD. Serum selenium concentrations were measured by instrumental neutron activation analysis. Odds ratios (OR) and confidence intervals (95% CI) for associations between serum selenium and ESD were calculated using unconditional logistic regression.

**Results:**

The mean serum selenium concentration was 85.5 (±28.3) μg/L. Forty-two ESD cases were identified (14% of those screened), including 5 (12%) in selenium quartile 1 (Q1), 5 (12%) in Q2, 15 (36%) in Q3, and 17 (40%) in Q4. Higher serum selenium was associated with prevalence of ESD (Q4 vs Q1: OR: 3.03; 95% CI: 1.05–8.74) and this association remained after adjusting for potential confounders (Q4 vs Q1: OR: 3.87; 95% CI: 1.06–14.19).

**Conclusion:**

This is the first study to evaluate the association of serum selenium concentration and esophageal squamous dysplasia in an African population at high risk for ESCC. We found a positive association between higher serum selenium concentration and prevalence of ESD, an association contrary to our original hypothesis. Further work is needed to better understand the role of selenium in the etiology of ESCC in this region, and to develop effective ESCC prevention and control strategies.

## Background

Esophageal cancer is the sixth most common cause of cancer death in the world, but its occurrence varies greatly in different geographic regions [1]. Esophageal cancer has two epithelial subtypes, esophageal squamous cell carcinoma (ESCC) and esophageal adenocarcinoma [2]. In many high income countries the incidence of ESCC has decreased, and is no longer the predominant form of esophageal cancer [3], but ESCC remains the dominant histologic type in several low and middle income countries where there are hot spot regions of high incidence [4–6].

East Africa is a region with high rates of esophageal cancer, a fact that has been known since the 1960s [7–9]. Data from the most recent Nairobi Cancer Registry found esophageal cancer to be one of the most common cancers among both men and women [10], and at Tenwek Hospital, in Bomet County in western Kenya, it is the most common cancer seen in both genders [11]. There is little published data on risk factors for ESCC in East Africa. Tobacco smoking and alcohol drinking account for 90% of ESCC in Western countries, but studies in other LMICs often report that these risk factors are much less important or absent [12, 13]. Low serum selenium has been reported to be an important risk factor for ESCC in the high risk regions of China [14, 15], but it has not been evaluated in Africa.

Selenium is an essential micronutrient in human nutrition that has been inversely associated with cancer risk in several previous studies, including one large prospective cohort study in China in which the low vs. high quartile attributable risk for ESCC incidence was 26% [14]. Similarly, low serum selenium levels were associated with an increased risk of developing ESCC and other cancers in large cohort studies in Finland and the Netherlands [16, 17]*.* Primary sources of dietary selenium include grains, leafy green vegatables, and animal products such as meat, fish, eggs, and dairy [18]. Soil selenium levels vary tenfold around the world, and the soil levels are reflected in locally grown plants and the serum of people who eat them [19]. Local soil selenium likely does not have much effect on populations such as those in the United States, where food supplies come from many different locations and are often transported long distances (an average of 1020 miles for final delivery of food) [20]. However, soil selenium can be important in areas where most dietary staples are locally produced and soil selenium levels are low [19].

The recommended Dietary Reference Intake for selenium from the Institute of Medicine is set at the amount needed to achieve a plateau in activity of the selenoprotein glutathione peroxidase [21]. Serum and plasma are the most useful tissues for assessing selenium status in humans, because selenium is stable in serum [22] and free of terrestrial contanimation. In serum, full activity of the selenoprotein glutathione peroxidase is reached at a selenium concentration of 90 μg/L [23], so this value was used as the threshold to assess selenium sufficiency in this analysis [24]. There are, however, additional benefits from the provision of selenium beyond this saturation point. A serum selenium level of around 120 μg/L has been shown to ensure optimal SePP saturation, which is important for distribution of selenium around the body [25].

The east coast of Africa has a high proportion of selenium deficiency based on regional food composition table estimates [26]. In Kenya over 90% of individuals are estimated to be at risk for selenium deficiency because they do not meet the estimated average requirement for daily intake [26]. A recent ecologic study which reviewed micronutrient deficiencies in the high esophageal cancer incidence countries in eastern Africa found a significant association between esophageal cancer incidence and low selenium nutrient supply [18].

A number of intervention trials have attempted to test the cancer preventive effects of selenium supplementation for esophageal squamous cell carcinoma [27]. In China, the Linxian General Population Nutrition Intervention Trial tested the effect of 5.25 years of daily supplementation with a combination of selenium, vitamin E, and beta carotene, and this intervention conveyed a significant reduction in the risk of developing gastric cancer, but no apparent effect on ESCC risk [28]. A 10-year post-intervention follow-up study showed a strong age interaction such that younger people at trial baseline had a lower risk of ESCC if they received this combined agent [29]. An observational analysis of this same trial cohort reported an inverse association between baseline serum selenium concentration and the incidence of ESCC [14]. This study also documented that the population in this region of China has low selenium status, with a mean serum selenium of 72.2 μg/L, which is well below the 90 μg/L threshold for sufficiency used in this study. A second randomized, placebo-controlled trial, conducted within the same high ESCC risk region of China, evaluated the effect of selenomethionine supplementation on the natural history of esophageal squamous dysplasia (ESD). ESD is the precursor lesion of ESCC and the only histopathology that predicts the development of ESCC [30]. This ESD supplementation study in China found increased dysplasia regression and decreased dysplasia progression among participants with mild dysplasia at baseline [31].

Overall, these studies from China suggest that selenium may play an important chemoprotective role in ESCC carcinogenesis in populations with low selenium status and high rates of this disease, possibly due to the action of selenium as an antioxidant [19]. Western Kenya is a high incidence area for ESCC, and it is known to have low soil selenium levels. The association of serum selenium status and ESCC prevalence has not been evaluated previously in this population. We measured serum selenium concentrations among Kenyan adult volunteers who were participating in a prospective clinical study assessing the prevalence of ESD in asymptomatic residents of southwestern Kenya [32].

## Methods

### Setting

**Fig. 1:**
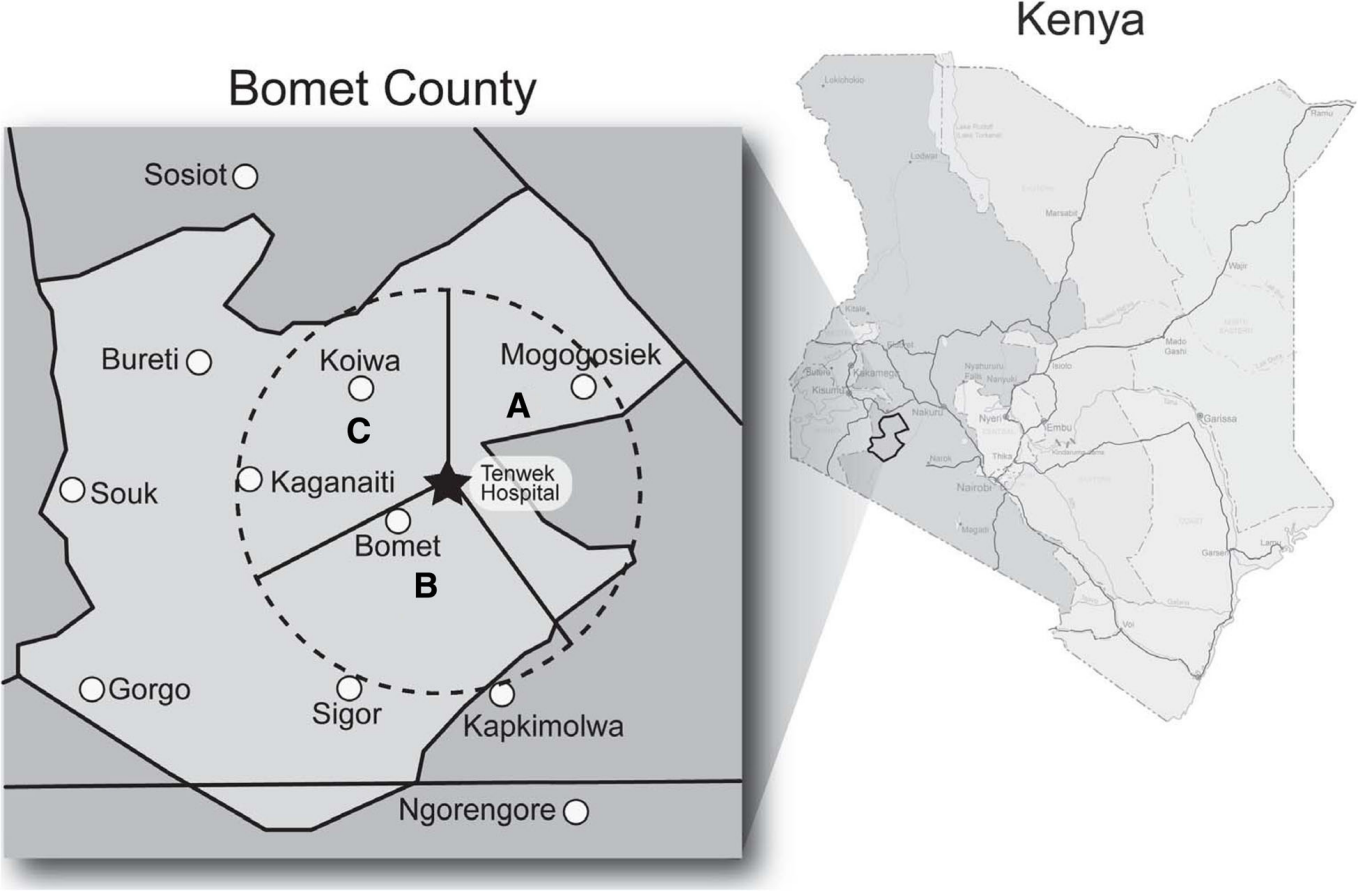
Tenwek ESD Study catchment area (dashed circle), which is divided into traditional zones labeled here as A, B, and C

Tenwek Hospital is a 300 bed mission hospital which serves as the primary health care facility for about 600,000 people. The 50 km radius of the Tenwek Hospital catchment area consists of three traditionally recognized geographic zones, which are displayed in Fig. 1. The zones vary by climate, agriculture, animal husbandry, and economic activity, but not ethnicity: Location A is characterized by fertile highlands with extensive tea and maize farms; Location B has dry sands with wheat farms and herds of cattle and goats; and Location C includes a mix of fertile and dry areas. Careful evaluation of differences in these three locations (in diet, soil selenium, etc.) have not yet been performed.

### Participants

Asymptomatic adults were recruited during 2010–2013 from within the Tenwek catchment area, including villages in each of the traditional geographic zones. Details of the recruitment procedures have been previously described [32]. Inclusion criteria were: residence within 50 km of Tenwek Hospital, age 20–79 years, and a signed informed consent. In total, 333 subjects were enrolled in the study. There were 20 missed appointments due to scheduling conflicts, and 313 individuals that arrived at the clinic for the study procedure. Subjects were specifically questioned about symptoms of dysphagia, odynophagia, hematemesis, or recent weight loss, and persons with any of these symptoms were excluded. Other exclusion criteria included allergy to iodine or lidocaine and pre-existing medical conditions that would increase the risk of endoscopy. Seven subjects were excluded based on these criteria. Of the 306 participants in the parent endoscopic study, 294 (96%) had both esophageal biopsies, to enable histologic diagnosis of dysplasia, and serum samples collected.

### Study approvals

Ethical approval for the parent endoscopic survey was obtained from the Institutional Review Boards of Tenwek Hospital and Kenyatta National Hospital in Nairobi, and all participants signed a written informed consent [32]. The selenium analysis was exempted from human subjects review by the National Institutes of Health Office of Human Subjects Research Protections (OHSRP).

### Questionnaire data

Trained study interviewers conducted in-person interviews with all ESD study participants. The questionnaire included questions about demographics, home indoor air pollution, occupational history, farming methods, diet, cooking and food preservation methods, medical history, family history of cancer, tobacco use, alcohol consumption, signs and symptoms of upper gastrointestinal disease, oral health, perception of anthropometry, and reproductive history (for female participants only).

### Blood samples

10 ml of venous blood was collected from all participants. Serum was separated, frozen at -20C, and shipped to the analytic laboratory on dry ice.

### Determination of serum selenium concentration

Serum selenium concentrations were measured by instrumental neutron activation analysis (INAA) at the University of Missouri Research Reactor. Samples were processed in three full run batches with the standard reference material National Institute of Standards and Technology (NIST) 909C. Three quality control (QC) samples were included in each batch along with one orphan run with a single QC sample. To generate the QC samples, serum from the 11 ESD study participants who did not have endoscopic biopsy results were pooled and then aliquoted into replicate daughter vials. 304 total samples were processed for this analysis, including the 10 QC samples. The measured Se value in 15 replicates of NIST 909C was 115 ± 9 μg/L and the certified value was 118.7 ± 3.3 μg/L. Among the QC replicates, the coefficient of variation for selenium was 10.5%. The limit of detection for the INAA method used in this study was 0.01 μg/g or 10 μg/L. The minimum selenium concentration measured in the study was 35 μg/L, and the 5th percentile was 47 μg/L.

### Endoscopic procedures

Esophageal squamous dysplasia is the asymptomatic precursor lesion of ESCC, and a strong predictor of ESCC progression [33, 34]. ESD can be visualized endoscopically after mucosal iodine staining with Lugol’s iodine solution, and then targeted for biopsy [30]. Participants in this study underwent endoscopy with Lugol’s iodine staining of the esophagus and biopsy of unstained areas ≥5 mm in size for detection of ESD. The endoscopy was conducted under conscious sedation (intravenous diazepam or midazolam and fentanyl), with standard vital signs monitoring and post-endoscopy care, and endoscopic findings were recorded using standardized data forms. Formalin-fixed, paraffin embedded biopsies were stained with hematoxylin and eosin and were used for histologic diagnosis using previously described criteria [35]. The biopsies were read at the Department of Human Pathology, University of Nairobi and at the National Cancer Institute, and discrepancies were adjudicated by joint consultation.

### Statistical analysis

Analyses were performed using STATA 13.0 software (StataCorp, College Station, Texas). Baseline characteristics were tabulated by serum selenium concentration. Significance of association was tested using a t-test for two-category and ANOVA for multiple-category variables. An alpha level of less than 0.05 was considered significant, and all tests were two sided.

**Fig. 2:**
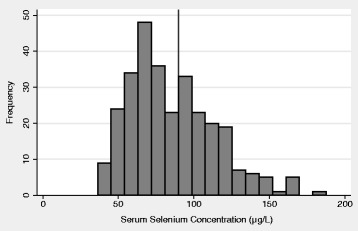
Histogram showing the distribution of serum selenium concentrations in the Tenwek ESD study population with the threshold of serum selenium sufficiency (90μg/L) noted

Serum selenium concentrations were approximately normally distributed across the 294 study participants (Fig. 2). For inclusion in logistic regression models as a continuous variable, selenium concentrations were scaled to half of the interquartile range ((Q3-Q1)/2 = 20 μg/L). Serum selenium concentrations were also analyzed categorically in quartiles. Because this was a cross sectional study in which all participants were asymptomatic, selenium quartiles were calculated based on all individuals in the study population. We undertook univariate analysis of demographic variables to assess the associations with serum selenium status. Odds ratios (OR) and confidence intervals (95% CI) for associations between serum selenium and ESD were calculated using unconditional logistic regression, adjusting for multiple potential confounders. *P for trend* values were assessed to determine the significance of the relationship between selenium and ESD in the quartile analysis.

Due to the lack of previous research, there were no assumptions about the relationship between selenium and ESD in the population of interest to include in the model a priori. Therefore, a crude model, an age- and sex-adjusted model, and a fully adjusted model were analyzed. The fully-adjusted model included the parameters in the previous analysis of the parent endoscopic screening study, which included adjustment for age (years), sex, education, residence location (A, B, or C), family history of cancer, family history of esophageal cancer, tobacco (regular use), and alcohol (regular use) [32]. Regular tobacco use was defined as at least once a day for at least six months, and regular alcohol use was defined as at least once a week for six months. As time of year may be an important factor for diet, month of study recruitment was also included to adjust for any potential effects due to seasonality.

Adjustment for other social class variables (income, number of household members, water source), medical history (heartburn, regurgitation, difficulty swallowing), other exposure risks (cooking indoors, animal contact), and dietary variables (ugali, fruit and vegetable consumption) was considered. A stepwise exploration to include all variables with *p* < 0.10 did not significantly change the selenium OR calculations, so these additional variables were not included in the final model. Stratification by category for each dependent variable was also conducted with the fully adjusted model to assess if there was any change in association between selenium concentration and histologically confirmed ESD. While results were slightly different when stratified by location, the differences were not significant and each location still demonstrated a positive association between ESD and serum selenium status, so these results are not shown.

## Results

294 participants had both biopsy and serum selenium results. There were nearly equal numbers of men (*N* = 158, 54%) and women (*N* = 136, 46%), as well as individuals age less than 50 years (*N* = 157, 53%) and greater than or equal to 50 years (*N* = 137, 47%). Of these, 174 study participants (59%) were below the 90 μg/L threshold used to assess selenium sufficiency in this study. Overall, the mean serum selenium concentration was 85.5 ± 28.3 μg/L, and the median serum selenium concentration was 79.4 μg/L (IQR: 63.9 μg/L-103.7 μg/L). Forty-two ESD cases were identified (14% of those screened), including 5 cases (12%) in selenium quartile 1 (Se Q1), 5 (12%) in Se Q2, 15 (36%) in Se Q3, and 17 (40%) in Se Q4.

Table 1 presents the distributions of patients, mean serum selenium concentrations, and significance of association with selenium for all ESD study participants across baseline characteristics. Serum selenium was found to be strongly associated with location of residence (*p* < 0.001). The mean selenium concentrations in location A (70.6 ± 19.6 μg/L) and location C (81.5 ± 22.9 μg/L) were significantly lower than the mean concentration in location B (118.8 ± 25.8 μg/L). The occurrence of ESD was also significantly associated with selenium concentration (*p* = 0.02). Participants diagnosed with dysplasia had a higher mean serum selenium concentration (94.9 ± 29.0 μg/L) than participants who were diagnosed as normal or esophagitis (83.9 ± 28.0 μg/L).

**Table 1:** Tenwek ESD study serum selenium concentration (μg/L), by participant characteristic

Characteristic	Participant n (%)	Serum Selenium Mean (SD)		*p* value^*^
Sex				
Male	158 (54)	85.3	(25.6)	0.94
Female	136 (46)	85.6	(31.2)	
Age				
< 50	157 (53)	83.8	(29.0)	0.29
≥ 50	137 (47)	87.3	(27.5)	
Residence				
Location A	91 (31)	70.6	(19.6)	*p* < 0.0001
Location B	58 (20)	118.8	(25.8)	
Location C	144 (49)	81.5	(22.9)	
Education				
Primary or greater	100 (34)	84.6	(25.8)	0.70
Less than primary	194 (66)	85.9	(29.5)	
Family history of cancer				
Yes	33 (11)	89.8	(30.0)	0.35
No	261 (69)	84.9	(28.1)	
Family history of esophageal cancer				
Yes	18 (6)	84.5	(27.4)	0.88
No	275 (94)	85.5	(28.5)	
Tobacco				
Regular Use^a^	56 (19)	85.8	(25.7)	0.91
No regular use	238 (81)	85.4	(28.9)	
Alcohol				
Regular Drinking^b^	92 (31)	83.0	(24.4)	0.31
No regular drinking	201 (69)	86.7	(30.0)	
Esophageal Squamous Dysplasia (ESD)				
Dysplasia	42 (14)	94.9	(29.0)	0.02
No Dysplasia	252 (86)	83.9	(28.0)	

^*^T-test for two-sample and ANOVA for multiple samples

^a^Regular use defined as at least once a day for at least 6 months

^b^Regular drinking defined as at least once a week for 6 months

The associations of continuous or categorical measures of serum selenium and ESD using unconditional logistic regression are shown in Table 2. Results are shown for the crude model, the age- and sex-adjusted model, and a model further adjusted for six other potentially confounding variables. In the continuous analysis, scaled to half of the interquartile range (20 μg/L), every unit increase in serum selenium concentration was significantly associated with ESD in all three models (fully adjusted model OR: 1.42; 95% CI: 1.04–1.93). In the quartile analysis, the association of selenium concentration and ESD increased from the lowest to the highest quartile of serum selenium in all models (p for trend ≤0.02), but only in the highest quartile of serum selenium was the association statistically significant (fully adjusted model OR: 3.87; 95% CI: 1.06–14.19).

**Table 2:** Odds Ratios, 95% CI, and *p* values for the association between serum selenium and prevalent ESD in the Tenwek ESD study

Scaled Serum Selenium^a^	OR	*95% CI*		*p* value				
Crude Model	1.29	1.04–1.61		0.02				
Age and Sex Adjusted	1.30	1.04–1.63		0.02				
Fully Adjusted^b^	1.42	1.04–1.93		0.03				
Selenium Quartile	Q1	Q2		Q3		Q4		
Mean Concentration (μg/L)	53	68		87		122		
Range (μg/L)	36-61	62-73		74-99		100-188		
ESD Cases, N (%)	5 (12)	5 (12)		15 (36)		17 (40)		*P* for trend
	*Ref*	OR	*95% CI*	OR	*95% CI*	OR	*95% CI*	
Crude Model	1	1.02	0.28–3.71	2.42	0.83–7.04	3.03	1.05–8.74	0.01
Age and Sex Adjusted	1	1.02	0.28–3.72	2.25	0.76–6.66	2.95	1.02–8.55	0.02
Fully Adjusted^b^	1	1. 13	0.29–4.37	2.71	0.84–8.69	3.87	1.06–14.19	0.02

^a^Selenium scaled to ½ Interquartile range (20 μg/L)

^b^Adjusted for age (years), sex, education, location (A, B, or C), family history of cancer, family history of esophageal cancer, tobacco (regular use), and alcohol (regular use)

## Discussion

We found a significant positive association between higher serum selenium concentration and prevalence of ESD, the precursor lesion of ESCC, which was contrary to our original hypothesis. Previous studies have found a significant inverse relationship between serum selenium concentration and ESCC risk [14], so this unexpected result should be interpreted with caution [36]. The relationship between serum selenium concentration and ESD may truly be different from the relationship between selenium and ESCC, but this seems unlikely.

It is also unlikely that this result was due to reverse causation, since all participants were asymptomatic, so their disease probably did not affect their eating habits. We think it is more likely that the unexpected findings of this cross-sectional study may be the result of length biased sampling [37]. If selenium is protective against ESCC carcinogenesis, as prior literature suggests, then it is possible that subjects with higher selenium status may have slower progression of ESD, which would make subjects with ESD more likely to be sampled in a cross-sectional screening study. In this case, prospective studies may still show an inverse relationship between serum selenium concentration and odds of developing ESD.

Selenium is an essential element that is necessary in small amounts for human health [38], but at a high dose selenium becomes toxic and may even promote carcinogenesis [39]. In the United States Nutritional Prevention of Cancer trial there was no protective effect of selenium supplementation among participants whose baseline plasma selenium concentrations were in the upper tertile of baseline plasma selenium (>121.6 μg/L) [40]. The protective effect was most prominent in those in the lowest tertile at baseline (≤105.2 μg/L). More research is needed to better understand how selenium may act differently in individuals with different nutritional status [38]. However, it seems clear that the population observed in this study is on the low end of the selenium distribution.

This study is noteworthy because it provides documentation that serum selenium status in this population is low. The threshold used to assess selenium sufficiency in serum in this analysis was 90 μg/L [24]. The majority (59%) of participants in this study fell below this threshold. The mean serum selenium concentration was 85.5 μg/L, which was higher than the 72.2 μg/L mean selenium concentration found in the high risk population of Linxian, China [14], but was significantly lower than the 136.7 μg/L mean concentration found in the third National Health and Nutrition Examination Study (NHANES) in the United States [41]. Fewer than 6% of individuals in the ESD study population had serum selenium concentrations higher than the NHANES mean value.

Maize grain is likely the primary determinant of selenium intake in this region [42]. Many residents grow their own maize in small plots around their homes, and use this locally grown maize to make their own ugali (the dietary staple in this population). Thus, differences in soil selenium in the area could be reflected in serum selenium status. Unfortunately, our study did not collect the data needed to evaluate these possibilities, and neither the soil selenium nor the selenium content of ugali have been measured in this area. In our analysis, there was no correlation found between ugali intake and serum selenium. Increased ugali consumption may be an indicator of lower socioeconomic status, in which case the most important effect of eating more ugali may be *not* eating a more varied healthy diet (which could also include other selenium sources), rather than being a direct predictor of serum selenium status.

Our study has a number of notable strengths. It is the first study to look specifically at the relationship between serum selenium and the presence of ESD in a population in a high ESCC incidence region in Africa. All patients in this prospective, population-based study were interviewed directly, which allowed for complete data collection. Our study used the best available method (Lugol’s chromoendoscopy, biopsy of unstained areas, and histologic diagnosis) to identify ESD status, and we directly measured serum selenium concentration in blood samples, in contrast to estimating individual selenium availability from dietary data. However, our study was limited by its relatively small size, with only 42 cases of dysplasia (33 mild, eight moderate, and one severe), and the fact that we measured selenium concentrations at only one time point, which might not be broadly representative of general selenium status.

## Conclusion

This is the first study to evaluate the association of serum selenium concentration and esophageal squamous dysplasia in an African population at high risk for ESCC. We found a significant positive association between serum selenium concentration and ESD, an association contrary to our original hypothesis. More work needs to be done to understand the role of selenium and other risk factors in the etiology of ESCC in this region, and to develop effective preventative and control measures.

## Abbreviations

CI: Confidence interval
ESCC: Esophageal squamous cell carcinoma
ESD: Esophageal squamous dysplasia
INAA: Instrumental neutron activation analysis
IQR: Interquartile range
LMIC: Low and middle income country
NCI: National cancer institute
NHANES: National health and nutrition; examination study
NIH: National institute of health
NIST: National institute of standards and technology
OHSRP: Office of human subjects research protections
OR: Odds ratio
Q: Quartile
QC: Quality control
SD: Standard deviation
